# Personalizing mHealth Interventions for Occupational Stress: Protocol for a Randomized Pilot Study

**DOI:** 10.2196/68012

**Published:** 2025-06-03

**Authors:** Beatrix Kunas, Oliver Jung, Christoph Schranz, Mathias Schmoigl-Tonis, Manuela Ploessnig, Anton-Rupert Laireiter

**Affiliations:** 1 Department of Psychology Paris-Lodron University Salzburg Salzburg Austria; 2 Salzburg Research Forschungsgesellschaft mbH Salzburg Austria; 3 Independent Researcher

**Keywords:** mHealth, occupational stress, personalized apps, multilevel diagnostics, just-in-time adaptive intervention, artificial intelligence

## Abstract

**Background:**

Occupational stress is associated with detrimental consequences that are addressed by mobile health (mHealth) solutions. Previous developments of apps for occupational stress have not yet fully exploited the potential of multilevel diagnostics through the integration of wearable sensors for interventions. Personalizing mHealth approaches in terms of intervention time and content, which requires the use of artificial intelligence, is the next logical developmental step. The "Relax" approach developed a corresponding prototype of an app-wearable system, which will be evaluated for effectiveness in terms of stress reduction and usability.

**Objective:**

This study protocol describes an evaluation study used to test the effectiveness and usability of the Relax approach.

**Methods:**

The evaluation study was designed as a 2-arm randomized trial with 2 phases, each with a 3-week intervention period. In both phases, employees were required to use the app to record daily stress and to wear a wearable sensor to measure heart rate variability. The app offered interventions based on algorithms, which altered the probability of their selection after learning from the data, thereby personalizing the user experience. In the second phase of the study, the sample was divided into 2 groups, varying the degree of personalization of the app. To analyze effectiveness, a 2-factorial mixed within-between design will be applied to compare the outcomes between both groups as well as in a pre-post comparison. In addition, exploratory analyses of the usability of the approach are planned.

**Results:**

The study was conducted during the spring and summer of 2024, with a total of 46 participants enrolled, and is ready for data analysis.

**Conclusions:**

The Relax approach, including a number of factors related to personalization that have not yet been incorporated into mHealth in current research, will provide new insights into the next steps of advanced mHealth solutions. Limitations of the study design, such as the lack of a control group and the sample representativity, have to be addressed.

**Trial Registration:**

Open Science Foundation 10.17605/OSF.IO/MYRD9; https://osf.io/myrd9

**International Registered Report Identifier (IRRID):**

DERR1-10.2196/68012

## Introduction

### Occupational Stress

For employees, work has the potential to be the most significant source of stress in their life. The working environment, which is often characterized by deadlines, role requirements, uncertainties, and time constraints, can be particularly stressful [1]. When stress is perceived as matching one’s abilities and motivations optimally, it can be referred to as “eustress” or a “flow experience” [2] and is characterized by positive emotions and high performance [3]. The term “distress,” which will henceforth be referred to simply as “stress,” describes the harmful type of stress that cannot be easily managed by the individual. Chronic work-related stress has been demonstrated to have detrimental impacts on an individual’s emotional well-being and overall quality of life [4]. Loss of energy and efficiency, mental and physical strains, and absence from work owing to burnout or other forms of mental health impairments are common consequences of unaddressed stress [5-7]. The study by Nekoei et al [8] recorded the suspected economic costs attributable to burnout as >2% of the national labor income, which demonstrates a continuous increase in workloads and speaks of an “epidemic of burnout” [9] affecting all fields of occupation, although some of them, such as health care, school education, or geriatric care, are particularly affected [10,11].

The provision of health care for working people with regard to stress-related burdens remains fragmented. The study by Molek-Winiarska and Molek-Kozakowska [12] showed that less than half of the organizations they had addressed committed to provide stress management support for their employees. In recent years, there has been a notable increase in interest in digital health solutions [13]. The COVID-19 pandemic, which placed many employees in unanticipated stressful work conditions, has demonstrated the urgent need for person-centered health care in the occupational setting, which can be effectively delivered by digital means [14]. The domain of mobile health (mHealth) addresses this type of individual health care, wherein mobile apps are used to facilitate cost-efficient and low-threshold access to effective interventions [15]. As the smartphone can serve as a constant companion during working hours [16], the provision of close health support via smartphone apps is particularly advantageous.

A plethora of mHealth apps addressing work stress are available in commercial sectors, as well as in scientifically based products [17-19]. The apps in question are highly diverse and exhibit considerable variation in their efficacy. Most apps concentrate on microinterventions derived from cognitive behavioral therapy (CBT), encompassing relaxation techniques, meditation, psychoeducation, and cognitive methods [17-19]. It may take many trial-and-error attempts for an employee to select a suitable approach [20]. An offer from the employer themself would be a logical and straightforward solution, particularly given the individualized nature of work stress, which can vary considerably depending on the specific workplace context. Given the continued tendency to regard mental health issues as a taboo subject in professional contexts [21], it is evident that mHealth apps for occupational stress are typically addressed at the individual level through the use of the employee’s personal smartphone rather than through the direct involvement of the employer. However, these apps must be tailored to the specific needs of the individual employee in terms of the stress-inducing circumstances and modes of coping and intervention without being set up by the employer. Therefore, the customizability of the app is important in developing an mHealth app for individual occupational stress.

### Personalized mHealth for Work Stress

The relevance of personalized apps that provide interventions for occupational stress is emerging [22,23]. On the one hand, personalization can be achieved simply through the input of personal data, which is then processed by the apps to customize interventions. To illustrate, a user may personalize the app using demographic data or specify the notification behavior for the intervention offer [24,25]. In addition to developments in the area of personal input and settings, there have also been significant advances in recent years in the field of combining apps with more advanced diagnostic measurement tools. The technical advancements in the domain of mobile sensors, or “wearables” for short, have opened up the opportunity for unobtrusive measurement of individuals’ current conditions through the use of wearable devices [26,27]. In the realm of stress diagnostics and interventions, this is particularly advantageous, as it enables the unobtrusive determination of heart rate variability (HRV), which is defined as a significant and practical physiological indicator of stress, although not the only one [28,29].

A multilevel diagnostic approach, which incorporates subjective input and objective sensor data, can facilitate the development of mHealth solutions for occupational stress management [17,26]. Such solutions are personalized on the basis of real-time data, a process referred to as “just-in-time adaptive interventions” (JITAIs) [30]. In this context, both the timing of the intervention and the content of the intervention are adapted to the situation and individual and therefore are necessarily linked to real-time data from the individual [31]. The necessity for the integration of multilevel data, coupled with the considerable volumes of data amassed through the integration of apps and wearables, necessitates the involvement of algorithms and learning agents in the background [32]. The development of artificial intelligence (AI) has led to its adoption in numerous domains of human-machine interaction, with the objective of enhancing user experience and personalizing processes [33]. In addition to smart technology such as voice assistants, the deployment of AI in health care apps could pave the way for the development of personal health assistants.

### Recent Developments and Research

This approach to health assistants is still in its infancy in terms of research and product development. General mHealth interventions with unobtrusive wearables are on the horizon, and their effectiveness has already been confirmed by several studies [34,35]. However, there is still a lack of attention being paid to the specific field of occupational stress [26,36]. Some approaches focus on safety systems that use wearables to monitor physical risks such as temperature and analyze physical stress but do not take mental stress into account [37]. Other approaches that explicitly examine mental stress have yielded the development of numerous systems designed exclusively for monitoring one’s own stress levels [27]. These systems lack the capacity for active interaction, such as the implementation of interventions. A recent review of the literature reveals that although mobile apps and wearable devices for mental stress interventions are also being developed, in most cases, they are based on either one or the other tool [26]. Combinations of both tools, in which the evaluated data are dynamically integrated, remain a barely addressed approach.

mHealth biofeedback apps provide an opportunity for such interactions, thus facilitating personalized mHealth interventions for occupational stress. In this approach, some developments have used real-time sensor data to feed into the app, which then provides the user with feedback on their stress levels [38]. When such measurements and feedback are coupled with breathing interventions, users have the ability to influence their heart rate through controlled or guided breathing [22,39-41]. Such interaction with the app allows for personalized data processing and can be seen as a basic form of individualization. There was only one approach that offered other CBT interventions in addition to biofeedback units for work stress, but these did not use the sensor data [22], which means that the full potential of continuous condition monitoring is not realized. Another way to personalize interventions is to use personal (sensor) data to determine the content of the intervention, a module, or the timing of the intervention, according to the JITAI concept. This idea was realized in the approach by Winslow et al [42], where CBT interventions were selected according to previous stress indications and conceptualized in the studies by Clarke et al [43] and Luštrek et al [44].

When developing such approaches, it is important to consider their real-world applicability as potential health companions in the everyday lives of their users. The feasibility of such apps goes beyond the technical capabilities and is closely related to usability factors. A review of the literature on mHealth usability shows that privacy is a particularly important concern for mHealth users [45,46]. This is evidenced by the fact that users express discomfort with the digital storage of sensitive health data, such as cardiac data or irregularities, within nontransparent commercial products [47]. A lack of motivation to integrate mHealth approaches into personal routines could be a consequence. Motivation, acceptance, and adherence issues are recognized as challenges in the development of mHealth [48,49]. In addition, although developed for the general population, highly educated, younger, or middle-aged individuals might access and adopt them more frequently, thus showing another challenge in the development of usable systems [50].

### The "Relax" Approach

The "Relax" approach involves the development of a system for multilevel diagnostics of occupational stress, with the aim of using these data to personalize interventions. This approach addresses the shortcomings of existing developments in the domain of personalized mHealth for work stress and extends the status quo by gathering a broad range of data for an AI-driven system to identify the most personally appropriate interventions from a diverse pool of contemporary CBT and health approaches.

The system combines a smartphone app for monitoring both direct and indirect subjective stress-related data, as well as dispositional personal data, and wearable devices for the assessment of cardiovascular indications of stress. The subjective data were multilayered, with dispositional and demographic characteristics serving as the basis for an individual profile for further personalization, as well as continuous data about current experiences that the AI would use to gain insight into the individual’s daily life. The data obtained from the sensors covered the area of objective stress measurement. The agent, trained with personal and stress-related data, was not only to determine the timing of an app-delivered intervention on an individual basis. In addition, the agent should select an individually tailored intervention that is as appropriate as possible at this predetermined point in time. Thus, the Relax project was planned to address the research on the development of JITAIs in mHealth for occupational stress in an advanced way. The concept of this approach, with its complexity and AI-driven personalization in the context of psychological support for work stress, addresses the existing gap in the field.

However, at the time of the evaluation, the system had not yet reached the final stage of development, that is, not all planned technical functions had been integrated. Regarding just-in-time quality, the functionality of the trigger system was limited to the essential function of triggering an intervention at a predetermined threshold of personal stress. This intervention has its adaptive quality in that the participants had to navigate through a predefined decision algorithm, in the form of a decision tree, when a stress event was detected to be offered an appropriate intervention in terms of situational fit. The specific probability of selection could be influenced and thus personalized by the participants through feedback ratings.

The pathways of the tree were represented by a series of successive questions about the experience of the current stress event. These were answered personally, resulting in increasingly specialized questions until, at the end, an intervention was delivered that was suitable for the input. The possible interventions from the repertoire were not exclusive per pathway, allowing for a wide range of interventions per pathway with different probabilities. The system decided which single intervention to offer the user based on the probabilities. The probabilities could be influenced by how the participants rated the intervention after it had been presented. Consequently, following a sufficient period of use, the probabilities of the interventions should be strongly modified on an individual basis, thereby reflecting the personal preferences for the interventions that are perceived to be personally most effective.

### Research Questions

This study aims to evaluate the prototype developed to date. The central research question of this evaluation is the effectiveness of the prototype in terms of the amount of stress reduction achieved by the participants. It is hypothesized that the greater the degree of personalization offered, the greater the reduction in stress. In addition, it was expected that all individuals would more or less experience an overall reduction in stress as a result of using the prototype. In addition to effectiveness, questions of usability and feasibility were addressed because of the pilot nature of the project approach and the research stage of the prototype at the time of the study. No hypotheses were formulated here, as the corresponding data were collected for exploratory purposes.

## Methods

### Study Design

The study used a 2-arm randomized controlled design, comprising 2 experimental groups with varying levels of intervention. In a field design, participants applied the intervention in their natural (occupational) environment. Two 3-week study periods were conducted, with all participants receiving the same intervention in the first 3-week phase (P1) and, after a 3-week break, being allocated to 1 of 2 groups in the second 3-week phase (P2). This resulted in a within-subject comparison in the pre-post design as well as between-subject comparisons between both experimental groups. The within-subject design consisted of the collection of time series data enabled by the continuous daily measurement of variables. Subjective data on experiences and stress levels were collected daily in the morning and evening, as well as at least twice during the day, via the app. Continuous physiological data were also collected via wearing a sensor. In contrast, the between-subject design comprised 3 measurement points: before P1 (T1), between P1 and P2 (T2), and after P2 (T3). At these times, web-based questionnaires about chronic stress and various usability aspects of the wearable app system were submitted via a web-based survey platform. The break was used to improve the mobile app based on user feedback, split the participants into the experimental groups, train the models for automated stress detection based on the HRV and questionnaire data recorded in P1, and implement the weight adjustment mechanisms for personalized intervention delivery.

### Ethical Considerations

The study design and conditions were initially approved in advance by the Ethics Committee of the University of Salzburg (EK-GZ 25/2023). Following minor alterations to the study design, including adjustments to the study duration, study period, the exclusion of a second sensor, and the reduction of the number of participants, an amendment was approved by the same committee (EK-GZ 25/2023 amendment). After data collection, a preregistration for hypothesis testing was performed (osf.io/myrd9). Due to the unpredictable nature of the study and the technical adjustments required during implementation, prospective preregistration was not performed.

Before completing the screening questionnaire, respondents were informed of the purpose of the data collection in order to provide informed consent. Those who were screened positively were informed of the acceptance of their registration via the email address they had provided. Those who were screened negatively were informed of the exclusion immediately after the screening. After the conclusion of the registration period, the final registration confirmations were sent out to all individuals included in the optimally balanced sample. Subsequently, the positions of those who had dropped out before the commencement of the study were filled by new participants until shortly before the study began. The identities of the participants were pseudonymized before the collection of study data. Participants were provided with an individual, reproducible code comprising questions on personal characteristics that could not be traced back [51]. The identity was only traceable by means of a coding list, which was kept by the study management and was only consulted in serious technical cases, dropouts or when checking whether the level of participation was sufficient to apply for the proposed compensation. Interested individuals were informed about the financial compensation worth €180 (US $204.86) (including the value in kind of retaining the study sensor). They could opt out at any point without negative consequences.

### Study Participants

The sample consisted of individuals who were of legal age and employed. Participants were required to possess a smartphone with a minimum Android operating system (version 7.0). In advance of the study, participants were required to demonstrate a sufficient level of knowledge of the German language, equivalent to that of a native speaker, as well as normal or corrected-to-normal eyesight. To be eligible for inclusion in the study, participants were asked to indicate that they had sufficient time to use the app daily during the study period, including having the opportunity to use the app every 3 to 4 hours. In addition, participants were only eligible if they had a regular working day that was not interrupted by holidays, significant changes in employment status, or travel during the study period.

Individuals who met the following criteria were excluded from the study: those with irregular work routines during the study period (eg, irregular night shifts), cardiovascular diseases, or atypical menstrual cycle complaints in women, those with upcoming medical interventions during the study period, and those with a diagnosed severe mental disorder or undergoing psychotherapy or psychopharmacotherapy. Inclusion and exclusion criteria were clarified by self-report unless the information was obviously evaluable. There were no restrictions regarding the location of participants in German-speaking countries or their occupational fields. The participants were able to communicate with the study team via online correspondence, including email and telephone.

### Sample Size

The optimal sample size for the central analysis of main effects was calculated using an a priori power analysis with G*Power (version 3.1) [52]. With an α value of .05, a power of 1-β=0.80, and a desired effect size of Cohen *d*=0.5, this resulted in a recommended sample size of 31. The sample size was increased in the study to the maximum number of participants that could be financed, which was 50 individuals. This resulted in a maximum of 25 (50%) individuals per experimental group.

### Recruitment

The ad hoc sample was constituted by recruiting potential participants via a variety of parallel channels. The recruitment process was centered on a study website that advertised participation and provided access to the study registration portal. First, precommitted companies were contacted and reminded of their participation. These companies were provided with the necessary information and access data for the registration portal for their employees. In addition to the previously committed companies, further companies were contacted and selected ad hoc from web-based registers. The professional network of the members of the study team was also addressed. Moreover, the public was made aware of the study through the dissemination of flyers in public spaces and on social and traditional media platforms. These flyers were placed in locations frequented by the public, including public areas and local businesses, as well as distributed via direct mail in the region of Salzburg and neighboring German towns. Dedicated social media accounts were set up on Facebook (Meta Platforms, Inc) and Instagram (Meta Platforms, Inc), which used public relations strategies that extended beyond the dissemination of study-related information, such as sharing posts on general information on stress management, to achieve a broader reach. The study accounts were used to contact relevant social media groups, including those of employees and individuals with an interest in research or stress. In addition, individuals who regularly posted on work-related topics were also contacted. Similarly, articles on the Relax project were published in traditional regional and national newspapers with a specific reference to the study.

The registration portal directed respondents to a web-based screening that inquired about the inclusion and exclusion criteria, as well as basic demographic data, including age, gender, and current employment sector. The collection of demographic data was undertaken to ensure the resulting sample was representative of the target population.

### Randomization

The randomization process was conducted midway through the study, following the scheduled break. No data from P1 were used to balance the randomization in any way. The participants were randomly assigned to the groups *local* and *global*. For the group *local*, the intervention weight matrix was primarily affected by participants’ own ratings, while the ratings of all participants affected the weights for the group *global*. The group *local* was regarded as the group with greater personalization than the group *global*, given that the personalization in question was based on the individual inputs.

The participants were not informed of their group allocation, which meant that they were considered blinded. As the randomization was conducted by a separate subgroup of the study team from those involved in the study itself and the data analysis, the latter 2 parts of the study can also be described as double blinded. The evaluation of the data in pseudonymized form further enhanced this aspect of independence.

### App Intervention

The intervention consisted of the use of the system of the Relax app and the wearable sensor developed in the Relax project. The participants were encouraged to use the system daily in P1 and P2. To accommodate the planned technical adjustments that will be made throughout the course of the study, all participants will be required to commence the study and complete the phases simultaneously.

The Relax app was developed based on a predefined mock design at the kickoff of the Relax project. This design was crafted with future projects in mind, ensuring long-term usability for data collection across several subsequent studies. The data management plan adhered to the European Union regulations. Bluetooth Low Energy (Bluetooth SIG) facilitated communication between the device and the phone, while REST interfaces enabled data exchange between the phone and the database.

The app interface consisted of several areas: the dashboard, the questionnaires, the interventions, calendar page, support page, user information, and an overview of the sensor data (provided in P2). In the dashboard, participants could check the status of the connection and synchronization of the sensor and monitor their study’s progress. For the questionnaires, profile and daily questionnaires were available. The profile questionnaires consisted of a series of 7 diagnostic measurements about living and working conditions, technological competence, work-related and everyday stressors, risk factors, resources, coping strategies, and consequences of stress. The profile data were designed to be administered in the first week of P1 to serve as a source of personal information for personalizing the interventions in subsequent weeks of the study. However, due to technical barriers, this information could not be used for further personalization.

The daily questionnaires consisted of morning and evening questionnaires and interval questionnaires during the day. The morning questionnaires included items on the quality of sleep during the previous night, current state and experiences and expected stress for the day. The evening questionnaire included questions about chronic stress, the current state and experiences, and summarized experiences of the day. The interval questionnaires asked about the current state and experiences and the state and experiences at the time of the last stressful event, if any. Participants were required to complete the morning and evening questionnaires and at least 2 interval questionnaires each day. They received daily reminders for the morning and evening questionnaires. The interval questionnaires were to be completed approximately 4 hours apart at the latest. This interval ensured that the questionnaires could be completed smoothly, even on participants’ busy working days. Participants were reminded several times a day to complete the interval questionnaires: twice in P1 (noon and 3 PM) and 3 times in P2 (11 AM, 2 PM, and 5 PM).

In addition to the daily use of the app, the sensor was to be worn to record physiological stress. The Polar Verity Sense (VS; Polar Electro OY), a wristband photoplethysmography sensor with an integrated inertial measurement unit from which various HRV parameters can be derived, was used for this purpose. The sensor was to be worn day and night in P1 and P2. Participants were instructed to take a maximum of two 30-minute breaks per day to avoid overstimulation and recharge the battery. It was possible to limit the duration of use individually if they felt uncomfortable, for example, to take it off at night. None of the participants took up this offer.

From the second week of the study in P1, interventions were triggered on the basis of individual stress information. On the one hand, a trigger was immediately released when (very) high stress was subjectively noted within the daily questionnaires. On the other hand, a trigger was released from objective sensor data. Multiple HRV metrics were calculated from measured sensor data based on 5-minute intervals. A stress intervention was triggered if (1) both the root mean square of successive differences (RMSSD) and the low frequency (LF) to high frequency (HF) ratio exceeded a predefined absolute threshold (deceded for RMSSD; exceeded for LF/HF ratio), (2) both metrics were among the 25% most significant values for the individual within a 24-hour window, and (3) both conditions held for 2 consecutive 5-minute time intervals.

From the trigger, a notification displayed in each area of the app provided access to the decision tree, which used various questions about the circumstances of the current stress experience to offer the optimal individual intervention. The main areas of the decision tree were external stressors, internal stressors, and indirect stress-related areas. For external stressors, the focus was on a dichotomy between occupational and other stressors, while for other stressors, it was noted that the Relax project only addressed occupational stress, so the participants were redirected to the top level of the decision tree. Internal stressors were divided into amplifiers, emotions, reactions, and resilience. The peripheral areas were divided into prevention and further information. All of these areas were further specified by the participants through up to 4 levels of predefined pathways. At the end of each path, there were several possible interventions.

The possible interventions were weighted differently depending on the end point of the pathway. The weights ranged from 0 to 1, with a higher weight increasing the likelihood of allocation. In the preliminary stages of the project, the initial weights were set according to the expert opinion of the development team. After P1, the initial weights for intervention selection were adapted based on all available user interactions. Popular intervention for a specific context received increased weights, thus a higher probability of being chosen in the subsequent context. Within P2, the weights of these mappings were adapted instantly for each intervention feedback.

The choice of intervention was made automatically by the system on the basis of the random weighted selection. At each node of the pathway, participants had the opportunity to immediately choose the weighted random selection of an intervention.

The 179 interventions were drawn from various areas of CBT, including psychoeducation, skills and attitude training, and relaxation and health habits. The interventions were designed as microinterventions that could be completed in a short period so that they could be easily integrated into everyday work life. Presentation formats varied across interventions depending on their applicability. These included simple text or tables with information, task prompts with a description and call to action, assisted tasks where participants received automated live instructions or otherwise interacted live with the app, or open prompts where participants were given an open field for response and self-reflection. In addition, there were pseudochats where participants were guided through automated educational chat transcripts, audio, or video content. At the end of an intervention presentation, the intervention could be rated. These ratings changed the initial weights of the intervention accordingly. Over time, this resulted in an individualized weight pattern of possible interventions for each person. For economic reasons, the system could not reach the stage at which interventions were offered fully automatically, so participants used the decision tree to receive interventions until the end of the study. Multimedia Appendix 1 illustrates the intervention areas and their subtypes in relation to their initial probability of occurrence with respect to the various stressor types within the decision tree. This higher probability is represented by the presence of an initial weight >0.5 (range from 0.0 to 1.0). In addition, Multimedia Appendix 1 depicts the presentation formats applied to these intervention areas and subtypes, as well as their quantities.

The level of individualization of interventions in the respective experimental group was characterized by a variation in automatic individualization with regard to the interventions offered by the learning agent. Due to intentionally high α values during the adjustment of the intervention weight matrix, the *local* group had the possibility of significantly influencing their intervention delivery within 3 weeks, while the group *global* would only perceive minor changes. α-local describes the extent to which a rating changed the weight of an intervention for a single user. For the group *global*, it was set to 1.0 (no change to the user’s own weight), and for the group *local*, it was set to 2.1. The weights were adapted using the following formula: new_weight=old_weight * (alpha_local^old_weight). α-global describes the extent to which a rating changed the weight of an intervention for all users. It was set to 21 for both groups. The weights were adapted using the following formula: new_weight=old_weight *(alpha_global^(old_weight/number_of_users)).

Data entry was continuously monitored by the study team throughout the weeks. Participants were encouraged to continue their engagement through regular emails from the study team. It was expected that a general increase in adherence would be achieved as these messages were sent to all participants equally. If technical problems occurred, they were addressed individually via the app and generally via email. It is assumed that such individual correspondences would not add to the overall bias of the user experience since the individual disruption was already a bias, which is not uncommon in pilot studies. In P1, an update of the app was offered in the second week, which solved the technical problems that had arisen up to that point. Participants were given support to reinstall the app, and a self-help guide for the most common technical problems was offered. During the break between P1 and P2, additional technical difficulties were overcome by the study team, and when feasible and relevant, the participants’ input thus far was considered. Following this adaptation of the system in accordance with feedback, a graphical overview of the sensor data was made available in P2. This enabled the participants to maintain awareness of the data and its evolution over time.

### Data Collection

#### Overview

Data for the study were collected from 2 sources: the mandatory app and wearable sensor data collected as part of the Relax approach and instruments implemented specifically for the study.

Items for the primary and secondary outcomes from established instruments were translated into German where necessary. Table 1 shows an overview of all outcomes, and Table 2 shows the data collection during the course of the study.

**Table 1:** Primary and secondary outcomes and their measurement conditions.

Outcome			Subscales		Design of measurement		Instrument		Item format		Example item
**Primary**											
	Momentary emotional state	Arousal, valence		Daily EMA^a^ (morning, interval)		Internally created scale		7-point Likert scale		“My current mood is:”	
	Emotional state of last stress event	Arousal, valence		Daily EMA (interval)		Internally created scale		7-point Likert scale		“My mood was:”	
	Chronic stress	Lack of satisfaction, high demand, loss of control, ability to recover, and negative emotions		Daily EMA (interval)		Selected items from CSSS^b^ [53] and LKCS^c^ [54]		7-point Likert scale		“I couldn’t relax today.”	
	Work-related distress	Relationship, personal accountability, work-life balance, and workload		Pre-post online		VEDAS^d^ [55], online		6-point Likert scale		“Dealing with ambiguous or ‘delicate’ situations.”^e^	
	Work-related eustress	Relationship, personal accountability, work-life-balance, and workload		Pre-post online		VEDAS [55], online		6-point Likert scale		“Dealing with ambiguous or ‘delicate’ situations.”^e^	
	Satisfaction	Overall satisfaction, workday adaptability, exertion due to EMA, and exertion due to sensor handling		Pre-post online		CSAT^f^ [56], internally extended), online		5-point Likert scale		“How would you rate your overall satisfaction with Relax?”	
	Usability	—		Pre-post online		SUS^g^ [57], online		5-point Likert scale		“I found using the Relax app unnecessarily complicated.”	
	HRV^h^	SDRR^i^, RMSDD^j^, and LF^k^/HF^l^		Continuously		Polar VS^m^		Passive		—^n^	
**Secondary**											
	Momentary activity	Environment (work or private), type of activity (if work: routine or extraordinary, if private: duty or leisure), physical exertion, mental exertion, and social activity		Daily EMA (interval)		Internally created scale		Dichotomous, 7-point Likert scale, single choice, open (text)		“Is work time right now?”	
	Activity of last stress event	Physical exertion, mental exertion		Daily EMA (interval)		Internally created scale		7-point Likert scale		“My physical exertion was:”	
	Expected stress	—		Daily EMA (morning)		Internally created scale		7-point Likert scale		“I expect for today:”	
	Subjective sleep quality	Subjective restfulness, sleep disruptions		Daily EMA (morning)		Internally created scale		7-point Likert scale, dichotomous, open (numeric)		“My sleep was:”	
	Summarized emotional state	Arousal, valence		Daily EMA (evening)		Internally created scale		7-point Likert scale		“My general mood throughout the day was:”	
	Summarized well-being	—		Daily EMA (evening)		Internally created scale		7-point Likert scale		“I felt overall today:”	
	Summarized activity	Environment (work/private), type of activity (if work: routine/extraordinary; if private: duty/leisure), physical exertion, and mental exertion		Daily EMA (evening)		Internally created scale		Dichotomous, 7-point Likert scale		“Has today been a workday?”	
	User feedback	Sensor handling, favorite function, requests on functionality, and general feedback		Pre-post online		Internally created scale		Open (text)		“What were your favorite features of the Relax app?”	
	Acceptable price	Optimal price, indifference price, marginal cheapness, marginal expensiveness		Pre-post online		PSM^o^ [58]		Open (numeric)		“At what price would the product on a monthly subscription basis be too expensive for you to buy it?”	
	Physical arousal	Mean HR^p^ and mean RR^q^		Continuously		Polar VS		Passive		—	

^a^EMA: Ecological Momentary Assessment.

^b^CSSS: Chronic Stress Screening Scale.

^c^LKCS: Leipzig Screening Questionnaire on Chronic Stress.

^d^VEDAS: Valencia Eustress-Distress Appraisal Scale.

^e^In the VEDAS, the same items were presented twice, once with a eustress framing of the instruction and once with a distress framing.

^f^CSAT: customer satisfaction score.

^g^SUS: System Usability Scale.

^h^HRV: heart rate variability.

^i^SDRR: SD of r-r-intervals.

^j^RMSSD: root mean square of successive differences.

^k^LF: low frequency.

^l^HF: high frequency.

^m^VS: Verity Sense. The same items were presented twice, once with a eustress framing of the instruction and once with a distress framing.

^n^Not applicable.

^o^PSM: price sensitivity meter.

^p^HR: heart rate.

^q^RR: r-r-intervals.

**Table 2:** Study schedule of enrollment, interventions, and assessments, according to the SPIRIT (Standard Protocol Items: Recommendations for Interventional Trials) guidelines [59].

Time point		Study period					
		Enrollment	Preallocation		Allocation	Postallocation	
		T0^a^	T1^b^	P1^c^	T2^d^	P2^e^	T3^f^
**Enrollment**							
	Eligibility screen	✓					
	Informed consent	✓					
	App installation	✓					
	Allocation				✓		
**Interventions**							
	App use			✓		✓	
	Sensor use			✓		✓	
	Microinterventions			✓		✓	
**Assessments**							
	VEDAS^g^		✓		✓		✓
	HRV^h^			✓		✓	
	Profile			✓			
	EMA^i^ (morning, interval, and evening)			✓		✓	
	CSAT^j^, SUS^k^, PSM^l^, and feedback				✓		✓

^a^T0: baseline.

^b^T1: before P1.

^c^P1: first 3-week phase.

^d^T2: between P1 and P2.

^e^P2: second 3-week phase.

^f^T3: after P2.

^g^VEDAS: Valencia Eustress-Distress Appraisal Scale.

^h^HRV: heart rate variability.

^i^EMA: ecological momentary assessment.

^j^CSAT: customer satisfaction score. The CSAT was expanded with 3 additional items.

^k^SUS: System Usability Scale.

^l^PSM: price sensitivity meter.

#### Primary Outcomes

First, primary outcomes were the emotional state over the course of the study and self-reported experience of chronic stress. On the one hand, levels of emotional states and stress were recorded daily using the ecological momentary assessment (EMA) technique. The data obtained from the daily subjective interval, morning, and evening interval questionnaires were used as a diagnostic tool. These questionnaires were developed internally, with established psychometric instruments used in certain cases. A team of experts was responsible for selecting the items for the internally developed instruments, some of which were commonly used items for the topics in question. Emotional state was assessed on the basis of the 2 dimensions: arousal and valence. In the interval questionnaire, in addition to the current state experienced at the time of entry, the state at a possible last stress event was also recorded in a similar manner.

The experience of chronic stress was evaluated using the evening questionnaire, which included items from the Chronic Stress Screening Scale [53] and the Leipzig Screening Questionnaire on Chronic Stress [54]. The Chronic Stress Screening Scale items assessed feelings of lack of satisfaction and high demands, while the Leipzig Screening Questionnaire on Chronic Stress items focused on experiences of loss of control, inability to recover, and negative emotions. The EMA questionnaires were each scored on a 7-point Likert scale. Conversely, chronic stress was evaluated using the Valencia Eustress-Distress Appraisal Scale [55] at the following time points after P1 and P2 in the web-based questionnaire. The questionnaire was used to distinguish between work-related distress and eustress, as well as to evaluate the subscales of relationship, personal accountability, work-life balance, and workload. A 6-point Likert scale was used in line with the original instrument.

Second, the physiological stress parameters, as measured by the Polar VS sensor, were recorded as primary outcomes. Similarly, the continuous data from the app use were used as an outcome. The stress parameters were HRV variables: SD of r-r-intervals, which is a direct measure of HRV; RMSSD, which is a measure of the parasympathetic recovery capacity; and LF/HF, which is the ratio of the balance between the sympathetic and parasympathetic nervous systems in favor of the latter.

Third, the level of satisfaction was assessed using the web-based questionnaire administered after P1 and P2. In this instance, the customer satisfaction score [56] was expanded with 3 supplementary items that were specifically designed to assess satisfaction and effort with the Relax approach. The items included ratings for overall satisfaction, workday adaptability, exertion due to EMA, and exertion due to sensor handling. The participants were required to indicate their level of agreement with the statements on a 5-point Likert scale.

Fourth, the System Usability Scale [57] was used to assess usability in the web-based questionnaire at P1 and P2. The items that were specifically reformulated for the Relax approach were collected using a 5-point Likert scale.

#### Secondary Outcomes

The interval questionnaire also included items on the momentary activities at the time of entry and the last stress event. The daily morning questionnaires for participants in P1 and P2 included additional items pertaining to expected stress levels for the day, as well as perceived restfulness of sleep from the previous night and the frequency and duration of sleep disruptions. The evening questionnaires inquired about the summarized emotional state along with well-being and the summarized activities of the previous day.

In addition to the quantitative survey, which was used as the primary outcome measure, satisfaction was also assessed using qualitative items. A total of 4 questions with an open item format were included in the survey to elicit feedback on the use of the app and the wearable device, as well as any suggestions or comments the participants might have. Finally, participants’ price acceptance was measured in the web-based questionnaire using the price sensitivity meter [58].

Within the displayed daily questionnaires, plain text fields allowed participants to report stressful events and special circumstances from the day. These responses were catalogued by the backend service and stored in the web-based database. However, they were not automatically analyzed by the system at this early stage of app development. Future research may explore automated analysis of stress-related text responses.

### Data Management

To prevent loss of the sensor data and ensure continuous 24/7 recording without gaps, a specialized workflow was designed for the app. All data were first stored locally on the VS system. Whenever a Bluetooth connection to the Android phone was established, a secure bulk transfer of all saved files was initiated, with a watchdog mechanism ensuring complete data reception. Once transferred to the phone, the data remained stored locally until an internet connection became available, at which point it was uploaded to the database in a similar manner as the Bluetooth transfer. If any data loss was detected, the entire file transfer process was restarted. Low battery charging problems had an inbuilt early detection system to prevent writing corrupted files on both systems.

The subjective data, pseudonymized using the self-generated code, were transferred to SPSS (version 29; IBM Corp) for data analysis. Means were calculated for all scales and then statistically analyzed. For the web-based questionnaires at T1, T2, and T3, exclusive values were created for the scales. The continuous daily data from P1 and P2 were transformed into weekly means for progression analysis. The broad time span of 1 week was intended to compensate for any missing data that were expected with complex EMA data collection integrated into daily life [60]. In addition, the general adherence to possible EMA entries and wearable data will be taken into account in the following statistical analysis. The records of individuals with at least >80% missing data will be excluded from the calculations. Data are available on reasonable request.

### Statistical Analysis

To statistically analyze the central evaluation questions of the prototype developed in the Relax project in terms of its efficacy in reducing stress in the workplace, different models will be constructed. First, the difference between the groups and thus the degree of individualization by the app will be determined. Second, the pre-post design will be used to ascertain whether the individuals exhibited a reduction in stress over time. These 2 approaches will use all stress-relevant outcomes to make assertions about the work stress of the individuals, namely the emotional states, self-reports on chronic stress as well as distress and eustress, and the physiological parameters. Linear mixed models will be used for the effectiveness analyses.

To address the usability and feasibility of the prototype, the data obtained from the satisfaction and usability scales will be used. These will also be analyzed between the groups and in the pre-post design. Exploratory analyses will test the secondary outcomes for possible effects. Additional moderation and mediation analyses will be possible here, in which the variables from the demographic and profile surveys could also be used. It is anticipated that the qualitative data from the user feedback will provide information about the specific background of the participants’ usability ratings.

## Results

The study was completed in the summer of 2024, and the statistical analyses are ready to be carried out. Data collection has reached a sample size of 46. Publication of the results is expected in the summer or fall of 2025.

## Discussion

### Anticipated Findings

The objective of the evaluation study of the prototype from the Relax project is to assess the effectiveness of the system, as well as its feasibility and usability. It is expected that the Relax approach will demonstrate stress reduction in working individuals according to comparable approaches in the field of mHealth for work stress [17]. However, this system occupies a distinctive position in the current research landscape, offering mHealth interventions in accordance with the concept of JITAIs, delivered at the optimal time and with content tailored to the individual for work stress management. Thus, it addresses the next step in development possibilities for current approaches that only focus on personalized interaction in individual interventions, such as biofeedback [22,39-41], or only make a rudimentary individualized module selection based on personal data [42]. The ability of a constantly learning app to select the optimal intervention for a given situation and individual from a pool of >100 interventions represents the potential of an AI-controlled mHealth app for occupational stress.

It is anticipated that, due to the pilot nature of the study, the results will be primarily dominated by the feasibility and usability aspects of the user experience. It should be noted that the app at the time of evaluation was in a raw version, which will inevitably result in a user experience that is not yet smooth. Furthermore, the Relax approach integrates a number of innovative design and development decisions, each of which represents a significant advancement in the field of mHealth for work stress [23,25]. The approach incorporates several novel elements, including AI-driven personalization, the implementation of the JITAI concept in both temporal and content aspects, and the incorporation of a multitude of potential interventions and continuous data input by the user. The results of the evaluation may provide insights into these aspects individually or generate new research questions regarding the design of advanced mHealth personalization and usability.

A special case is that, due to the individual personalization, the user experience may vary considerably from one individual to another, which can be challenging for an evaluation on a group basis. The fact that virtually every user could create a different experience in terms of the quantity and timing of the interventions can result in heterogeneous outcomes. Such heterogeneous results are desirable in analyses where individual personalization is a means to a general end, such as stress reduction or satisfaction with the app. In such cases, personalization must necessarily be heterogeneous due to the heterogeneity of personal stress experiences. However, such a heterogeneous user experience could lead to the app being perceived as unusable, for example, by users who experience little stress and therefore little interaction with the app.

### Limitations

The study design reveals some limitations in the significance of the results that are still to be analyzed. First, the pilot nature of the study permits only an initial impression of the effectiveness of the developed system in terms of stress reduction in an individual context. The absence of a control group without interventional procedure precludes certainty that any effects are undoubtedly attributable to the intervention. It is possible that the potential effects on stress reduction during the study period may be influenced by factors unrelated to the intervention. Consequently, a subsequent investigative study using a controlled design is imperative to clarify the causes of any effects.

Furthermore, the unspecified ad hoc sample represents a limitation. No distinction was made between professions and sectors, which means that user experiences can vary considerably not only due to individual characteristics and stress levels but also due to the specificities of individual everyday working lives. These can partly determine the degree of interaction with the app due to varying degrees of availability of the individual for using the app during working hours. It is therefore anticipated that adherence may vary considerably among individuals who are not able to adhere to the app use instructions and are unable to wear the wearable device continuously. The small sample size will not allow for subgroup analysis, as a highly variable sample is expected in this regard. However, it is also expected that the personalization approach of the app will adapt to the highly heterogenous work lives and still create the optimal experience for each individual user.

Another limitation of this study is that data collection was conducted exclusively on Android devices. This decision was driven by technical constraints, including iOS restrictions on background data access and sensor use, as well as the greater flexibility of Android for research applications. In addition, cost considerations and the higher prevalence of Android devices compared to iOS devices within our target population influenced this choice [61].

Technical challenges and adjustments, both planned and unplanned, can influence the behavior of the data. Technical issues may result in the absence of data recording during specific intervals or the occurrence of transmission errors. These factors have the potential to significantly distort use patterns and the overall picture of an individual’s stress levels. Further studies should incorporate a more advanced version of the prototype, informed by the feedback obtained in this study, with the objective of enhancing its usability and resilience to anticipated technical and adherence issues.

### Conclusions

The Relax approach provides a new perspective on how personalized elements can be incorporated into mHealth solutions, paving the way for more tailored and effective interventions according to the JITAI concept. This pilot study aims to test the developed system. The initial findings from this study will offer further insight into the feasibility of the system, as well as its preliminary effectiveness. However, there are several limitations to the study design that need to be considered, including the absence of a control group, concerns regarding the representativeness of the sample, and anticipated technical interference with data quality. Addressing these limitations is crucial for ensuring the robustness and generalizability of the results in future research.

## Appendix

Multimedia Appendix 1 Relationships between intervention areas, subtypes, initial weights, and presentation formats.

## Abbreviations

AI: artificial intelligence
CBT: cognitive behavioral therapy
EMA: ecological momentary assessment
HF: high frequency
HRV: heart rate variability
JITAI: just-in-time adaptive intervention
LF: low frequency
mHealth: mobile health
RMSSD: root mean square of successive differences
VS: Verity Sense
