# Supplementary material for: Personalizing mHealth Interventions for Occupational Stress: Protocol for a Randomized Pilot Study
Source: JMIR Res Protoc. 2025 Jun 3;14:e68012. doi: 10.2196/68012 (PMC12174872; doi:10.2196/68012)
Supplement: Multimedia Appendix 1 [file resprot_v14i1e68012_app1.docx]

Table S1. Relations between intervention areas, subtypes, initial weights and presentation formats.

| Intervention area | Subtype | N | Initial weights over 0.5 | | | | | | | Presentation format | | | | | | | | | | |
| --- | --- | --- | --- | --- | --- | --- | --- | --- | --- | --- | --- | --- | --- | --- | --- | --- | --- | --- | --- | --- |
|  |  |  | Work stress | Amplifiers | Emotions | Reactions | Resilience | Prevention | Info | | Text | Task | Assisted | Table | Open | Checklist | Chat | Audio | Video |  |
|  |  |  |  |  |  |  |  |  |  | |  |  |  |  |  |  |  |  |  |  |
| **Skills** |  |  |  |  |  |  |  |  |  | |  |  |  |  |  |  |  |  |  |  |
|  | Emotion regulation | 17 | x |  | x | x | x |  |  | |  | x | x |  |  |  | x | x | x |  |
|  | Time management | 13 | x | x |  |  |  |  |  | |  | x | x |  | x |  | x |  |  |  |
|  | Cognitive methods | 12 | x | x |  |  |  | x |  | | x | x |  |  | x |  | x |  |  |  |
|  | Social competence | 9 | x | x |  |  |  |  |  | |  | x |  |  | x |  |  |  |  |  |
|  | Conflict management | 8 | x |  |  |  |  |  |  | |  | x |  | x |  | x | x |  |  |  |
|  | Problem solving | 7 | x |  |  |  |  |  |  | |  | x |  |  | x |  | x |  |  |  |
|  | Selfcare | 7 | x | x | x |  | x |  |  | |  | x |  |  | x |  | x |  |  |  |
| **Psychoeducation** |  |  |  |  |  |  |  |  |  | |  |  |  |  |  |  |  |  |  |  |
|  | - | 42 | x |  |  | x | x | x | x | | x |  |  |  |  |  |  |  |  |  |
| **Attitudes** |  |  |  |  |  |  |  |  |  | |  |  |  |  |  |  |  |  |  |  |
|  | Positive psychology | 10 | x |  |  |  | x |  |  | |  | x |  |  | x |  |  |  |  |  |
|  | Work-related attitudes | 8 | x |  |  | x |  |  |  | |  | x | x |  |  | x | x |  |  |  |
|  | Values and goals | 7 | x | x |  |  | x |  |  | | x |  | x |  | x |  |  |  |  |  |
| **Health habits** |  |  |  |  |  |  |  |  |  | |  |  |  |  |  |  |  |  |  |  |
|  | Sleep | 10 |  |  |  |  |  | x |  | |  | x |  |  | x | x |  |  |  |  |
|  | Nutrition | 5 |  |  |  |  |  | x |  | |  | x | x | x |  | x |  |  |  |  |
|  | Environment | 5 | x |  |  |  |  | x |  | |  | x |  |  |  | x | x |  |  |  |
|  | Fitness | 4 |  |  |  | x |  | x |  | |  | x |  |  |  |  | x |  |  |  |
| **Relaxation** |  |  |  |  |  |  |  |  |  | |  |  |  |  |  |  |  |  |  |  |
|  | General techniques | 9 | x |  |  |  | x | x |  | |  | x | x |  | x |  | x | x |  |  |
|  | Emergency techniques | 7 | x |  |  | x |  |  |  | |  | x |  |  |  |  |  |  | x |  |
